# Chronicles of Pharmacy

**Published:** 1911-06

**Authors:** 


### Reviews of Books.

**Chronicles of Pharmacy.** By A. C. WOOTTON. Two Volumes. Pp. xii., 428; 332. London: Macmillan & Co. Ltd. 1910.

It cannot be said of the present age, as St. Paul said of the Athenians, that we spend our time in nothing else but either to tell or to hear some new thing, for simultaneously with the intense interest which modern discoveries excite there has grown up an eager desire to throw ourselves back into the past, to learn what our ancestors thought and did, and to trace to their very sources the springs of all our knowledge.

The two volumes before us deal with the chronicles of pharmacy from the dim dawn of early Egyptian mythology to the present age of great chemists and synthetic remedies. It is a matter of deep regret that the author did not live to see his work completed, and to enjoy the praise and welcome it deserves.

The volumes, which are well printed, have a good index and many illustrations. They cover a wide field, and deal with pharmacy not only in its strict meaning, but in its relation to medicine, toxicology, magic, and even poetry, and thus impart an interest which will be shared alike by the general reader and the specialist.

As the author himself says in the preface, "he has been tempted into various by-paths." In no case, however, is his information irrelevant, but it adds delight to profit, and the pleasant style enhances the value of the work. It has, moreover, the advantage that the chapters need not be read consecutively. They can be taken up in almost any order, and thus ensure to the busy man many a pleasant half hour. It appeals, as has been said, to a wide circle of readers. Thus the student of Shakespeare will find much to interest him in the chapter on "Shakespeare's Pharmacy," with its discussion on the nature of the "juice of cursed Hebenon," and the reference to the poet's son-in-law Dr. John Hall's method of practice. The biblical scholar will turn with interest to the chapter on the "Pharmacy of the Bible," which throws much light on the exact nature of many of the plants therein mentioned, and records the fierce controversies which have often been waged over their identity, the contention of St. Augustine that Jonah's gourd was the ivy having led from words to blows with his saintly antagonist St. Jerome. The folklorist and the historian, the chemist and physiologist, will be equally interested, and the wonder of the psychologist will be aroused at the extent of human credulity, for the sufferings of our ancestors at the hands of the surgeons in pre-anæsthetic days almost pale before their patient endurance in swallowing the loathsome remedies of the ancient pharmacists, many of whose concoctions can only be equalled by the contents of the witches' cauldron in *Macbeth.*

